# The Insurance Forum

**Published:** 1915-04

**Authors:** 


### The Insurance Forum.

April 3, 1914.

*Mrs. F. E. Daniel, Editor Texas Medical Journal, Austin, Texas.*

DEAR MRS. DANIEL: In line with our conversation of several days ago, I am sending you two, of the many, interesting letters received from the medical directors of life insurance companies doing business in Texas, in reply to the following inquiry directed to them: "Will you please tell me what your principal difficulties are in regard to the carelessness of your examiners relating to life insurance examinations?" This inquiry was the result of several strenuous complaints voiced by the medical directors as a result of the query as to whether their companies were paying the $5.00 fee for medical examinations.

Believing that the medical examiners as well as the medical directors of our State will be interested in this correspondence I

shall take the liberty of sending these letters to you from time to time for publication, and at the same time requesting that you omit the names and addresses of the companies and medical directors in copying, and return the original letters to me at your earliest convenience.

These letters are all from the medical directors of companies doing business in Texas, and whose companies are paying the flat fee of $5.00 for medical examinations. They clearly show that the medical directors of these companies, while favoring this fee, not only expect, but by right demand, prompt, honest, conscientious and loyally efficient service at the hands of the examiner.

These letters, of course, treat largely of the examiner's duties and relations toward the companies. I therefore take this method of requesting the examiners to write me fully their ideas regarding the relations of the companies toward their examiners, and beg, at the same time, to assure them that their letters will be held in strictest confidence should they so request. In this way we will be able to make a study of these questions from both standpoints, with a hope for our ideals of future perfect professional and business relations and confidence between the medical director and medical examiner.

Earnestly hoping that the perusal of these letters will stimulate some careless brother examiner to the best that is in him in his future work, not only in life insurance work, but that he may give that careful, loyal and efficient consideration to the patient who is entrusting him with his life and health as well. I respectfully submit them.

Sincerely yours,

M. B. GRACE.

---

December 8, 1914.

*Dr. M. B. Grace, Chairman Committee on Insurance, Seguin, Texas.*

DEAR DOCTOR: We have your favor of the 30th ultimo asking that we advise you with reference to the particular difficulties encountered in regard to the carelessness of examiners in making medical examinations, etc.

In reply, beg to say that if you refer to carelessness which makes it difficult to know what value to place on an examiner's recommendations, without further correspondence—the question of failure to make any investigation in regard to habits, or of not taking the matter of habits into consideration, even when they have knowledge of same—is probably our greatest difficulty, as a great many examiners simply record the statement of the applicant as given them, and where they have knowledge that it is incorrect, make no comment in regard to the matter in making up their recommendation of the risk.

Of course, any failure to answer questions or to give explicit information, which makes it necessary to return the examination causes more or less delay, but matters of this kind come up in so many ways that it would be difficult to say what particular omission causes the most trouble. The majority of examiners—or rather the majority of what we term careless examiners—do not make sufficient inquiry into family history, are careless as regards the question of measurements and in examination of urine.

We keep in this office a record of each examiner, showing temperature, pulse, chest measurements, specific gravity of urine, and the recommendation of the examiner as regards acceptance or rejection of the risk—and it would surprise you to note, even among our best examiners—that is, men that we know actually do their work thoroughly—to see the number of cases they will send in giving normal temperature, a standard pulse rate of 76, and four-inch expansion (difference between full inspiration and forced expiration) and specific gravity of urine as 1020.

While these items among healthy men should always run, or do run, somewhere around the normal, you will appreciate the fact that if taken accurately, could not always run exactly the same thing.

This, however, is carelessness only in recording specific data correctly, and means that there is no great departure from the normal—as where there is, the work of the examiner brings out the facts at the proper place in the examination and usually by comment under "Remarks."

Work such as you suggest, calling attention to the great number of examinations that have to be returned on account of unanswered questions, or insufficient data, would unquestionably be of great deal of advantage to the company.

---

*Dr. M. B. Grace, Seguin, Texas.*

DEAR DOCTOR: I am taking you literally at your word, that you want facts about local examiners for life insurance companies. Here they are! I have put the matter as straight as I know how to do it.

If there is any way on earth that you can get these ideas (clothed in your own words if you think best) before the medical profession of Texas, you will do a lasting favor not only to medical directors, life insurance companies, but to the local examiners themselves.

If I can serve you at any time in this matter I will certainly be glad to do so.

I may drop off and see you some time on one of my trips.

### POINTS TO BE NOTED REGARDING WORK OF LOCAL EXAMINERS FOR LIFE INSURANCE.

1. Carelessness has cost life companies more than ignorance or crookedness—a crooked examiner is very rare. So many examiners are content because they see a man daily for years and he has never been in bed sick to make a casual examination. The applicant may have albumen, heart murmurs, or incipient phthisis.

2. Few examiners seem to grasp what medical directors want. We wish all the information regarding the applicant, such as drinking and immoral habits, living in unhealthful surroundings, or in houses with tuberculosis.

3. Omissions. We estimate that in 30 per cent of the cases letters have to be written to examiners regarding omissions of answers, or to make clear certain points. For instance, the examiner answers "rheumatism," never saying whether muscular, sciatica or inflammatory. They will record "stomach trouble," never giving an idea whether it was a belch, an ulcer or a cancer. So many say "father" or "mother" dead, "don't know cause," when one parent is living in the same town and the desired information could be obtained in a moment.

4. Fifty per cent of the examiners record "pulse 72," "respiration 18," "temperature 98½," "urine 1020, acid, no sugar, no albumen." Now any physician knows something is radically wrong somewhere when this large number of cases are recorded as above.

5. When an examiner approves a case he virtually signs the company's demand note for $1000 and up. From this we can judge the gravity of a report of a thoughtlessly made examination of an applicant.

6. A great many examiners do not accommodate the agents by giving dispatch to business. Many an applicant will refuse an examination if delay is made; hence, since the fee is large enough, and payment prompt, quick service should be given.

7. There are many good examiners who love the work and do their full duty. One hundred per cent improvement has been made in the last four years, which speaks well.

8. The best service is gotten from the new graduate; they are taught now in medical schools about life insurance work, and, they need the money.

9. From the ranks of old busy practitioners, with big reputations and a tendency to surgery, come the poorest class of examiners.

10. We need education, publicity and agitation along these lines. The medical directors have an association, both State and national, whose members are trying to educate the doctors, inculcating all these points, that better service to the companies may result. The majority of examiners are responding, and medical directors feel pleased and fully repaid for the expense and trouble.

11. Examiners often forget that they are paid by the medical department and owe allegiance thereto, and not to the agent or the applicant. Courtesy is due the agent at all times, a square deal is due the applicant, and the benefit of the doubt is due the company.

12. Medical directors probably have not fully appreciated the worries and troubles of the examiners, and write them only when they make mistakes and omissions. The medical examiner should be written to and encouraged each time he does his full duty, or goes out of his way to serve the company. Medical directors are realizing this more fully, and adequate appreciation for good, safe, capable and honest examinations will, in future, be bestowed.

